# Genomic analysis of *Staphylococcus aureus* from the West African Dwarf (WAD) goat in Nigeria

**DOI:** 10.1186/s13756-021-00987-8

**Published:** 2021-08-19

**Authors:** Adebayo Osagie Shittu, Fadekemi Funmilayo Taiwo, Neele Judith Froböse, Bianca Schwartbeck, Silke Niemann, Alexander Mellmann, Frieder Schaumburg

**Affiliations:** 1 Department of Microbiology, Obafemi Awolowo University, Ile-Ife, Nigeria; 2 Institute of Medical Microbiology, University Hospital Münster, Domagkstraße 10, 48149 Münster, Germany; 3 Institute for Hygiene, University Hospital Münster, Robert-Koch-Straße 41, 48149 Münster, Germany

**Keywords:** *Staphylococcus aureus*, Ruminants, Goats, Whole-genome sequencing, Nigeria

## Abstract

**Background:**

*Staphylococcus aureus* can colonize various host species, and human-animal interaction is a significant factor for cross-species transmission. However, data on *S. aureus* colonization in animals, particularly on ruminants in close contact with humans, is limited. The West African Dwarf (WAD) goat is among the earliest domesticated ruminant associated with rural dwellers and small-holder farmers in sub-Saharan Africa. This study aimed to investigate the population structure, antibiotic resistance, and virulence gene determinants of *S. aureus* from the WAD goat in Nigeria.

**Methods:**

Nasal samples were obtained from the WAD goat in five markets in Osun State, South-West Nigeria. *S. aureus* was characterized by antibiotic susceptibility testing, detection of virulence determinants, *spa* typing, and multilocus sequence typing (MLST). Representative isolates were selected for whole-genome sequencing, biofilm, and cytotoxicity assay.

**Results:**

Of the 726 nasal samples obtained from the WAD goat, 90 *S. aureus* (12.4%) were recovered. Overall, 86 isolates were methicillin-susceptible, and four were *mecA*-positive (i.e., methicillin-resistant *S. aureus* [MRSA]). A diverse *S. aureus* clonal population was observed (20 sequence types [STs] and 37 *spa* types), while 35% (13/37) and 40% (8/20) were new *spa* types and STs, respectively. Eleven MLST clonal complexes (CC) were identified (CC1, CC5, CC8, CC15, CC30, CC45, CC97, CC121, CC133, CC152, CC522). The MRSA isolates were designated as t127-ST852-CC1-SCC*mec* type VII, t4690-ST152-CC152-SCC*mec* type Vc, and t8821-ST152-CC152-SCC*mec* type Vc. Phylogenetic analysis revealed that 60% (54/90) of all isolates were associated with ruminant lineages (i.e., CC133, CC522). Panton-Valentine Leukocidin (PVL)-positive *S. aureus* was identified in CC1, CC30, CC121, and CC152. For the CC522 isolates, we illustrate their pathogenic potential by the detection of the toxic shock syndrome gene and hemolysins, as well as their strong cytotoxicity and ability to form biofilms.

**Conclusions:**

This is the first detailed investigation on the genomic content of *S. aureus* from the WAD goat in Nigeria. The *S. aureus* population of the WAD goat consists mainly of ruminant-associated lineages (e.g., CC133, CC522), interspersed with human-associated clones, including PVL-positive MRSA CC1 and CC152.

**Supplementary Information:**

The online version contains supplementary material available at 10.1186/s13756-021-00987-8.

## Introduction

*Staphylococcus aureus* is a commensal inhabiting the skin and mucous membranes and a pathogen associated with a range of human and livestock diseases [1]. It colonizes various hosts mainly through genomic diversification and acquisition or loss of mobile genetic elements (MGEs), encoding immune evasion factors, leukocidins, and superantigens [2, 3]. The close contact between animals and humans and increased industrialization of livestock farming facilitate *S. aureus* host-switching and adaptation events [4]. *S. aureus* infections are of great concern in the dairy industry (comprising mainly cow, sheep, and goat) with economic implications [5]. Moreover, animals as a reservoir for *S. aureus* colonization portend serious consequences to human health [3].

From 2009 to 2013, an increase (18.3–42.3%) in the prevalence of methicillin-resistant *S. aureus* (MRSA) was observed with regional variations in Nigeria [6]. Also, studies based on two molecular typing schemes, i.e., *Staphylococcus* protein A (*spa*) typing and multilocus sequence typing (MLST), have provided evidence on some dominant clones. They include t064-CC8, t037-CC239 (MRSA) [7], and t084-CC15, t355-CC152 in methicillin-susceptible *S. aureus* (MSSA) [8]. Furthermore, the Panton-Valentine Leukocidin (PVL), a bi-component pore-forming toxin, is widespread among MSSA in CC121 [9, 10] and CC152 [8, 10]. Data on the molecular epidemiology of animal *S. aureus* in Nigeria is limited. Nevertheless, studies have observed a diverse *S. aureus* population, including human-associated lineages such as CC15 (PVL+) [11, 12], CC88 [12, 13], CC121 (PVL+), and CC152 (PVL+) [14] from food animals and their associated products.

The West African Dwarf (WAD) goat (*Capra hircus*) is a domesticated ruminant associated with humans and livestock farming in sub-Saharan Africa [15]. It is a major livestock resource, particularly among rural dwellers and small-holder farmers in West and Central Africa [16]. As of 2012, the goat population in Nigeria was 81 million, and it is estimated to reach 208 million by 2050 [17]. The close and long-standing interaction of the WAD goat with humans underscores the need to investigate possible *S. aureus* cross-species transmissions. The aim was to describe the clonal structure, antibiotic resistance, and virulence gene determinants of *S. aureus* in the WAD goat in Nigeria.

## Materials and methods

### Nasal sample collection

**Fig. 1:**
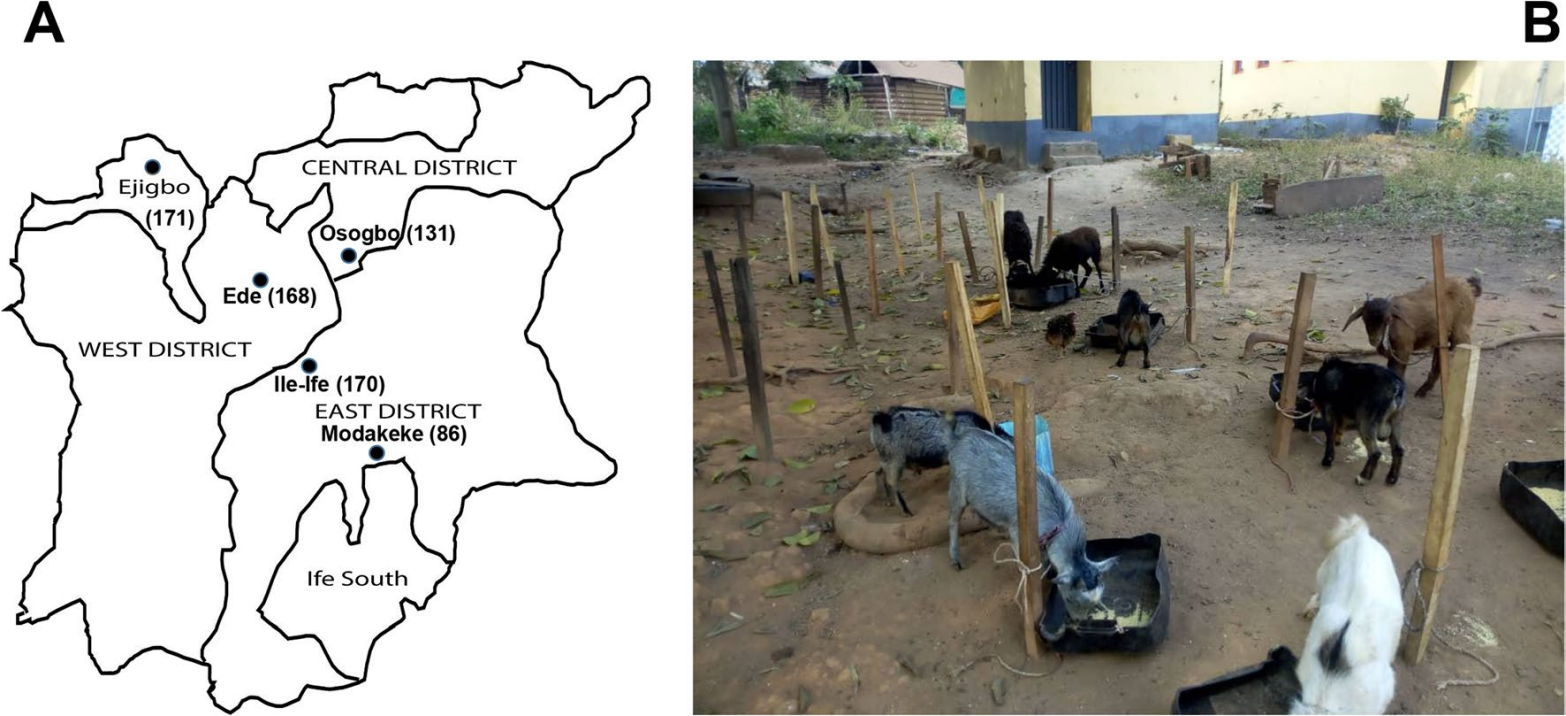
**a** The sampling of the WAD goats in markets located in five locations in Osun State, Nigeria. **a** Map of Osun State indicating the locations [62]. The number of nasal samples (in parenthesis) is indicated for each location. **b** The WAD goats in one of the markets

This study included WAD goats from the animal market located in five towns in Osun State, South-West Nigeria (Fig. 1a). The sampling period was from July 2018 to August 2019. A holding structure held the goats (Fig. 1b), and at each sampling event, all the animals in the custody of participating sellers were included. The subsequent visit to these markets was predicated on new animal stock information (provided by the sellers), thereby ruling out multiple sampling of individual goats. A nasal swab was taken using a sterile cotton swab stick (Sterilin, UK), moistened with sterile 0.85% NaCl solution, placed back to the swab pouch, and promptly transported to the laboratory. This step was followed by enrichment in nutrient broth (MAST Diagnostic, UK) overnight at 37 °C. Thereafter, 10 µl of the broth culture was streaked on Mannitol Salt Agar (MAST Diagnostic, UK) and incubated at 37 °C for 48 h.

### Characterization of *S. aureus* isolates

Preliminary identification as *S. aureus* was based on Gram staining and a positive catalase, coagulase, and DNase reaction. Isolates were confirmed as *S. aureus* by MALDI-TOF MS (Bruker Daltonics, Bremen, Germany), PCR detection of the *S. aureus* specific thermostable nuclease (*nuc*) [18], and nonribosomal peptide synthetase (*NRPS*) genes [19]. Methicillin-resistant *S. aureus* (MRSA) was confirmed by the detection of *mecA* [20]. Isolates were subjected to antimicrobial susceptibility testing (Vitek 2 automated system bioMérieux, Marcy l’Étoile, France) using EUCAST clinical breakpoints (Version 11.0), *spa* typing [21], and PCR detection of virulence (*lukS*/*lukF*-PV, *chp, sak*, *scn*) genes [22, 23].

### Whole-genome sequencing

One *S. aureus* representing each *spa* type (n = 37) was selected for whole-genome sequencing (WGS) on an Illumina MiSeq or NextSeq platform (Illumina Inc., San Diego, USA) with a 250-/150 bp paired-end protocol aiming for 100 × coverage [24]. Subsequently, reads were de novo-assembled using the SKESA assembler integrated into the SeqSphere^+^ software (version 7.0, Ridom GmbH, Münster, Germany). The antimicrobial resistance and virulence genes and multilocus sequence types (ST) were predicted in silico using SeqSphere^+^ as recently described [25]. The staphylococcal cassette chromosome *mec* (SCC*mec*) types of the MRSA isolates were determined by the SCC*mec*Finder 1.2 [26] from the Centre for Genomic Epidemiology (https://cge.cbs.dtu.dk/services/SCCmecFinder/; accessed on 22 April 2021). The raw reads of the representative isolates were deposited in the European Nucleotide Archive (https://www.ebi.ac.uk/ena) under the project accession number PRJEB44433. The Neighbor-Joining (NJ) tree was constructed using 1861 genes of the *S. aureus* core genome multilocus sequence typing (cgMLST) scheme (Task templates: *S. aureus* cgMLST v1.3, pairwise ignore missing values).

### Screening for hemolytic activity and PCR detection of hemolysin (*hla and hlb*) genes

One *S. aureus* of each *spa* type in the CC522 lineage (n = 9) was screened for hemolytic activity on Columbia sheep blood agar (CBA, BD, Sparks, MD, USA). Colonies were examined for hemolysis after CBA plates were stored at 4 °C for 24 h with an earlier overnight incubation at 37 °C [27, 28]. The presence of the hemolysin (*hla* and *hlb*) genes was also determined by PCR [29, 30]. *S. aureus* identified with an intact *hlb* (based on WGS) was also evaluated, and a positive and negative PCR result indicated a non-truncated or truncated *hlb*, respectively.

### Mucoidy and biofilm assay

Isolates representing the CC522 lineage were assessed for mucoidy on CBA. Two characteristics determined the criteria for mucoidy: (a) colonies stick tightly to the CBA plate and (b) colonies with chewing gum-like texture, as assessed by an inoculating loop, after overnight incubation at 37 °C [27]. Mucoid isolates were identified and further screened on modified Congo Red Agar (CRA) composed of brain heart infusion broth (37 g/l, VWR Chemicals BDH, Leuven, Belgium), bacteriological agar (15 g/l, VWR Chemicals BDH, Leuven, Belgium), sucrose (36 g/l, Neofroxx GmbH, Germany) and Congo Red (0.8 g/l, Waldeck GmbH & Co KG, Münster, Germany) [31]. The colony characteristics were noted after incubation at 37 °C for 72 h. To determine the amount of biofilm produced by the mucoid CC522 isolates, a static 96-well microtiter plate (MTP) assay was performed as described previously [32]. The absorbance of adherent biofilm cells was measured with a microtiter plate reader (Bio-Rad, Hercules, CA, USA) at 655 nm. Moreover, in parallel experiments, the nature of biofilms was analyzed. Biofilms were treated with sodium metaperiodate (NMP), which breaks down polysaccharide-mediated biofilms, and by proteinase K or DNase I, which disrupts protein- or DNA-dependent biofilms, respectively. Each mucoid *S. aureus* isolate was investigated in three biological replicates in eight wells per microtitre plate. Four isolates were used as controls: two biofilm-negative strains (*S*. *carnosus* TM300 and *S. aureus* 5bpdel^−^), a biofilm-positive strain (*S*. *epidermidis* RP62A (ATCC 35984) and the *S. aureus* CF-70518005-I (5bpdel^+^) that produces a biofilm, which consists of polysaccharide intercellular adhesin (PIA). PCR detection of the *icaA* and *icaC* genes was also performed, as previously described [32].

### Cytotoxicity assay

The representative CC522 isolates (n = 9) were evaluated for cytotoxicity. The toxicity of 20% bacterial supernatant (overnight culture in tryptone soy broth [TSB]) on A549, a human alveolar epithelial cell line (ACC 107, DSMZ, GmbH, Braunschweig, Germany), was determined after 24 h incubation using flow cytometry [33]. Cytotoxicity was indicated as the proportion of dead (i.e., % of propidium iodide [PI]-positive) A549 cells. Cells treated with 20% TSB served as control.

### Statistical analysis

The level of agreement between antibiotic susceptibility testing (AST) and detection of antibiotic resistance genes (WGS) was determined by Cohen’s κ test [34]. The κ test was also utilized to determine the level of agreement between PCR and WGS in detecting Panton-Valentine leukocidin (PVL) and immune evasion cluster (IEC) genes. The κ coefficient was interpreted as no agreement (κ < 0), slight agreement (κ: 0.00–0.20), fair agreement (κ: 0.21–0.40), moderate agreement (κ: 0.41–0.60), substantial agreement (κ: 0.61–0.80), almost perfect agreement (κ: 0.81–1.00) [34]. The analysis was performed using GraphPad Prism (https://www.graphpad.com/quickcalcs/kappa1/). In the static biofilm and detachment assay, results were indicated as means and standard deviation on GraphPad Prism 5.0 (GraphPad Software, Inc., San Diego, CA). A two-way ANOVA with the Bonferroni posthoc test was utilized to compare the absorbance values of the different groups (buffer, NMP, proteinase K, and DNase I) in the biofilm assay. In the cytotoxicity assay, the percentage of PI-positive mean values were analyzed using one-way ANOVA and compared with the negative control using Dunnett's multiple comparison test on GraphPad Prism. *P* < 0.05 was considered significant.

## Results

### Antibiotic susceptibility of *S. aureus*

Of the 726 nasal samples, 90 were positive for *S. aureus* (12.4%; one isolate per sample). All isolates were susceptible to glycopeptides, clindamycin, daptomycin, fosfomycin, fusidic acid, levofloxacin, linezolid, mupirocin, rifampicin, and tigecycline (Table 1). Moreover, 56% (50/90) were susceptible to all tested antibiotics (Additional file 1: Table S1). Four were identified as MRSA (*mecA*-positive). When comparing AST and WGS, a slight agreement was observed for fosfomycin and erythromycin (κ: 0.00). Substantial to almost perfect agreement was noted for gentamicin (κ: 0.65), tetracycline (κ: 0.72), penicillin (κ: 0.73) and oxacillin (κ: 1.00), respectively (Additional file 2: Table S2).

**Table 1:** Antibiotic susceptibility of *S. aureus* isolates from the WAD goat in Nigeria

	MSSA (n = 86)			MRSA (n = 4)		
Antimicrobial Agent	R	I	S	R	I	S
Penicillin	27	–	59	4	–	0
Oxacillin	0	–	86	4	–	0
Gentamicin	1	–	85	0	–	4
Levofloxacin	0	0	86	0	0	4
Azithromycin	1	0	85	0	0	4
Clarithromycin	1	0	85	0	0	4
Erythromycin	1	0	85	0	0	4
Clindamycin	0	0	86	0	0	4
Linezolid	0	–	86	0	–	4
Daptomycin	0	–	86	0	–	4
Teicoplanin	0	0	86	0	0	4
Vancomycin	0	0	86	0	0	4
Tetracycline	9	7	70	4	0	0
Tigecycline	0	–	86	0	–	4
Fosfomycin	0	–	86	0	–	4
Fusidic acid	0	–	86	0	–	4
Mupirocin	0	–	86	0	–	4
Rifampicin	0	0	86	0	0	4
Cotrimoxazole	6	2	78	3	1	0

MSSA: Methicillin-susceptible *Staphylococcus aureus*; MRSA: Methicillin-resistant *Staphylococcus aureus*; S, Susceptible; I, susceptible, increased exposure; R, Resistant

### Genotyping of isolates

**Fig. 2:**
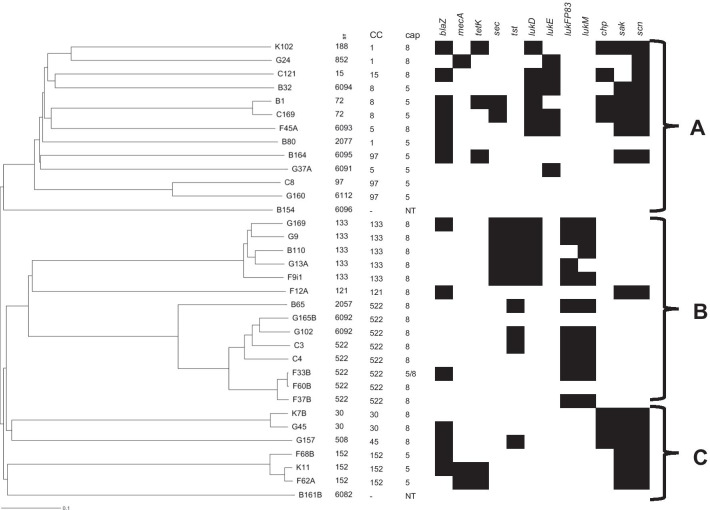
A Neighbor-Joining (NJ) tree of selected *S. aureus* isolates based on up to 1861 genes of the *S. aureus* core genome (cg)MLST scheme and annotated with Clonal complex (CC), capsule type, and antibiotic and virulence gene carriage. ST, Sequence type; CC, clonal complex; cap, capsule; Antibiotic resistance genes and product (*blaZ*, β-lactamase; *mecA*, alternate penicillin-binding 2a; *tetK*, tetracycline efflux protein variant K). Enterotoxins and toxic shock syndrome genes (*sec*, enterotoxin C; *tst*, toxic shock syndrome toxin). Leukocidin and hemolysin genes (*lukD*, leukocidin D; *lukE*, leukocidin E; *lukF-P83*, bovine Panton-Valentine leukocidin subunit F; *lukM*, leukocidin M). Immune evasion cluster genes (*chp*, chemotaxis-inhibiting protein; *sak*, staphylokinase; *scn*; staphylococcal complement inhibitor) □ negative ■ positive

The isolate collection consists of 37 *spa* types, including 13 new ones. MLST identified 20 STs with eight new STs (Table 2). Overall, the predominant *spa*-CC types comprised t3576-CC522 (n = 15), t10018-CC522 (n = 12), t18949-CC97 (n = 7), t9268-CC522 (n = 6) and t18947-CC133 (n = 4). The MRSA isolates were classified as t127-ST852-CC1-SCC*mec* type VII (n = 2), t4690-ST152-CC152-SCC*mec* type Vc (n = 1), and t8821-ST152-CC152-SCC*mec* type Vc (n = 1, Table 2; Additional file 3: Fig. S3). Based on the construction of the NJ tree, the isolates were assigned into three main clusters (Fig. 2). Cluster A was a divergent group consisting of CC1, CC5, CC8, CC15, CC97, and ST6096. Cluster B is made up of closely related ruminant-associated lineages comprising CC133 and CC522, including CC121. CC30, CC45, CC152, and ST6082 were grouped with cluster C.

**Table 2:**
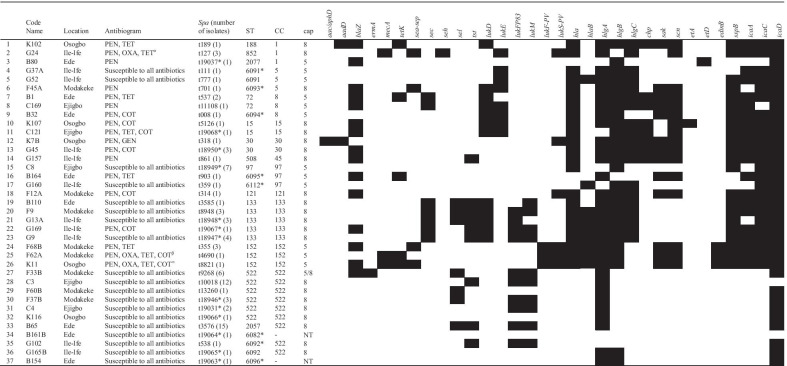
Characterization and detection of selected antibiotic and virulence genes (WGS) in *S. aureus* isolates from the WAD goat in Nigeria

PEN, Penicillin; GEN, Gentamicin; OXA, Oxacillin; TET, Tetracycline; COT, Trimethoprim-sulphamethoxazole; *spa*, *Staphylococcus* protein A; ST, Sequence Type; CC, clonal complex; cap, capsule; Antibiotic resistance genes/product (*aacA-aphD*, bi-functional aminoglycoside phosphotransferase; *aadD*, aminoglycoside adenyltransferase; *blaZ*, β-lactamase; *ermA*, rRNA adenine N-6-methyl-transferase gene; *mecA*, alternate penicillin-binding 2a; *tetK*, tetracycline efflux protein variant K). Enterotoxins and toxic shock syndrome genes (*sea*-*sep*, enterotoxin A and P; *sec*, enterotoxin C; *seh*, enterotoxin H; *tst*, toxic shock syndrome toxin). Leukocidin and hemolysin genes (*lukD*, leukocidin D; *lukE*, leukocidin E; *lukF-P83*, bovine Panton-Valentine leukocidin subunit F; *lukM*, leukocidin M, *lukF-PV*, Panton-Valentine leukocidin subunit F; *lukS-PV* Panton-Valentine leukocidin subunit S; *hla*, hemolysin alpha; *hlaB*, hemolysin B; *hlgA*, hemolysin gamma component A; *hlgB*, hemolysin gamma component B; *hlgC*, hemolysin gamma component C). Immune evasion cluster genes (*chp*, chemotaxis-inhibiting protein; *sak*, staphylokinase; *scn*; staphylococcal complement inhibitor). Exfoliative toxin and epidermal cell differentiation genes (*etA*, exfoliative toxin A; *etD*, exfoliative toxin D, *edinB*, epidermal cell differentiation inhibitor B). Proteases (*sspB*, staphopain B). Biofilm associated genes (*icaA*, intercellular adhesion gene A; *icaC*, intercellular adhesion gene C; *icaD*, intercellular adhesion gene D) □ negative ■ positive; *new *spa* types and sequence types; ^α^: t127-ST852-CC1-SCC*mec*VII; ^β^: t4690-ST152-CC152-SCC*mec*Vc; ^∞^: t8821-ST152-CC152-SCC*mec*Vc

### PCR detection of PVL and IEC genes

PVL-positive *S. aureus* was identified in CC1 (n = 2), CC30 (n = 4), CC121 (n = 1) and CC152 (n = 5) including MRSA in CC1 (n = 2) and CC152 (n = 2). Furthermore, PCR detection of the IEC genes showed that isolates in CC30 and CC45 were *chp*/*sak*/*scn*-positive including CC1 (n = 2), CC8 (n = 2), and CC152 (n = 1, Additional file 1: Table S1). CC15 isolates were uniquely *chp*/*scn* positive, while those assigned with CC1 (n = 2), CC5 (n = 1), CC8 (n = 1), CC97 (n = 1), CC121 (n = 1), CC152 (n = 3) were *sak*/*scn* positive. The IEC genes were not detected in CC133, CC522, ST6082, and ST6096 including *S. aureus* in CC1 (n = 1), CC5 (n = 2), CC8 (n = 1) and CC97 (n = 1). We observed a high level of agreement (κ: 0.9–1.00) between PCR and WGS in the detection of the above-mentioned IEC genes, while moderate agreement (κ: 0.55) was noted for the detection of the PVL gene (Additional file 4: Table S4).

### Antibiotic resistance genes and *S. aureus* lineages

WGS and MLST showed that *blaZ* conferring beta-lactam resistance was identified in all CCs. However, most of the isolates in CC133 and CC522 were *blaZ*-negative (Table 2). Only one CC30 isolate with phenotypic resistance to gentamicin was positive for the corresponding resistance (*aac-aphD* and *aadD*) genes. The *tetK* was the gene determinant for tetracycline-resistant *S. aureus* in CC1, CC8, CC97, and CC152 (Table 2).

### Capsule typing, detection of virulence genes, and hemolytic activity

**Fig. 3:**
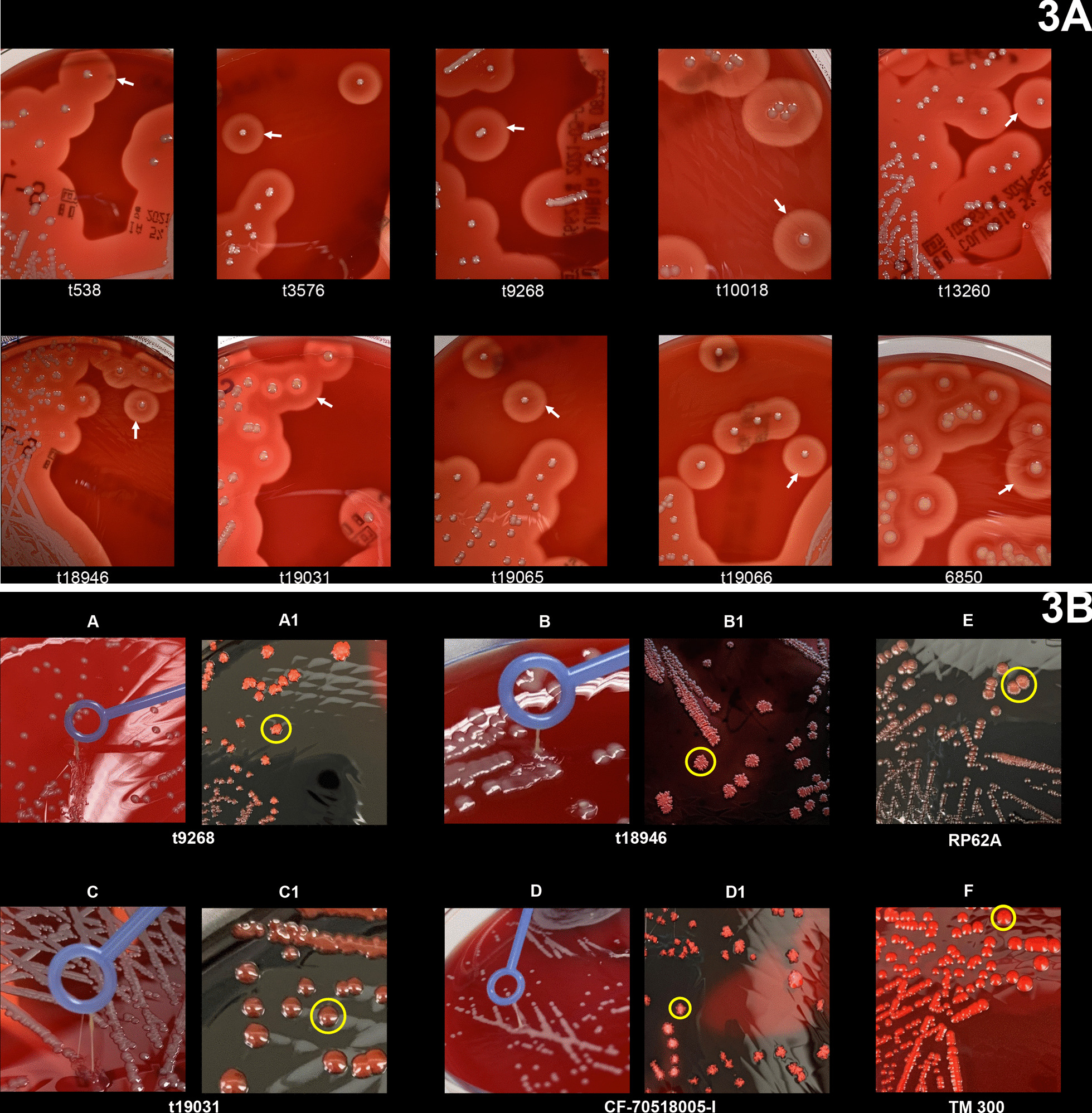
**a** Hemolytic activity of representative CC522 *S. aureus* isolates on Columbia Blood Agar (CBA). Positive control: highly invasive and cytotoxic strain *S. aureus* 6850. Colony sizes might differ due to different camera positions. The zone of hemolysis (β-hemolysis) is indicated with an arrow. The *spa* types of representative CC522 *S. aureus* are presented. **b** Mucoid CC522 isolates on CBA and CRA. Key: A: Columbia Blood Agar (CBA); B: Congo Red Agar (CRA). A, B, C, D: mucoid phenotype; A1, B1, D1, E: pink/brown colonies with wrinkled, irregular edges; C1: brown colonies with smooth, convex, entire edges; F: pink colonies with smooth, convex, entire edges. Positive control: RP62A—*S. epidermidis* (biofilm-positive); 70518005-I: cystic fibrosis (CF) *S. aureus* isolate with a 5 bp deletion within the intergenic region of the *ica* operon [32] (biofilm/PIA-positive); Negative control *S. carnosus* TM300 (biofilm-negative). The *spa* types of mucoid CC522 *S. aureus* are indicated. Colony morphology is highlighted, and sizes might differ due to different camera positions

WGS of selected *S. aureus* (n = 37) representing each *spa* type established that capsule type 5 was associated with CC1, CC5, CC8, CC97, and CC152. Capsule type 8 was identified with CC1, CC5, CC15, CC30, CC45, CC121, CC133, and CC522 (Table 2). The *sea-sep* genes were detected in CC1, CC5, and CC152. Only one CC1-MRSA was *seh*-positive. The distribution of the enterotoxin genes and clonal lineages revealed that *S. aureus* isolates in CC45, CC133 (*sec*-positive), and CC522 were *tst*-positive. Moreover, the bi-component leukocidin (*lukF-P83*/*lukM*) genes and *sel* were detected only in CC133 and CC522 (Table 2). Also, some CC5, CC8, and CC15 isolates were positive for *lukDE*. The majority of isolates (62%, 23/37) carried *hla* across the lineages, but this gene was not detected in CC522 by WGS (Table 2). However, representative CC522 isolates exhibited strong hemolysis on CBA (Fig. 3a). PCR confirmed that these isolates harbor *hla* and *hlb* (Additional file 5: Fig. S5). WGS also revealed that some *S. aureus* in CC5, CC97, CC133 (*chp*/*sak*/*scn*-negative), and CC152 (*sak*/*scn*-positive) carried the intact *hlb* gene and confirmed by PCR. The complete *hlg* operon comprising *hlgA*, *hlgB* and *hlgC* was identified in *S. aureus* assigned with CC1, CC5, CC8, CC15, CC30, CC45, CC121, and CC133. The exfoliative toxin (*etA* and *etD*) genes were detected in CC15 (n = 1) and CC1 (n = 1), respectively and only CC152 isolates were *edinB*-positive.

## MSCRAMMs genes and screening for mucoid phenotype and biofilm assay

**Fig. 4:**
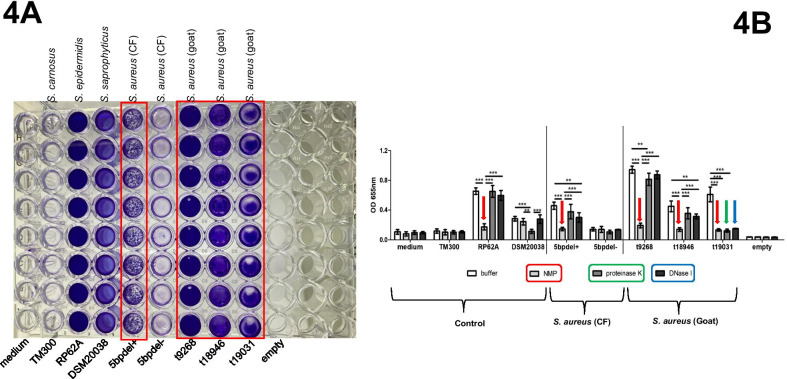
**a** and **b** Semi-quantitative biofilm and detachment assay of CC522 *S. aureus* isolates. TM300: *S. carnosus* (biofilm-negative); RP62A: *S. epidermidis* (biofilm/PIA-positive); DSM20038: *S. saprophyticus* subsp. *saprophyticus* (biofilm/protein-positive); 5 bp^+^: *S. aureus* isolate (CF-70518005-I) with a 5 bp deletion within the intergenic region of the *ica* operon; 5bpdel^−^: *S. aureus* isolate (CF-70518005-II) without the 5 bp deletion in the intergenic region of the *ica* operon; t9268, t18946 and t19031: *spa* types of *S. aureus* isolates from the WAD goat. CF: Cystic Fibrosis; NMP: Sodium metaperiodate. Bars represent the mean and standard deviation of three independent experiments. Statistical significance: *p*-value < 0.01 (**); *p*-value < 0.0001 (***)

The most common protease gene was *sspB*, and WGS identified the intercellular adhesion (*icaA* and *icaC*) genes in > 80% of isolates across the various CCs but absent in CC522 (Table 2; Additional file 6: Table S6). Also, PCR could not detect some portions of the *icaA* and *icaC* genes in representative CC522 isolates. However, t9268, t18946, and t19031 *S. aureus* in CC522 exhibited the mucoid phenotype on CBA. Besides, t9268 and t18946 isolates displayed rough colonies with wrinkled edges, while t19031 exhibited round, convex brown colonies on CRA (Fig. 3b). The MTP assay showed that all three isolates are biofilm producers. However, the biofilms attached to the bottom of the MTP appeared different from those produced by the PIA-positive *S. aureus* CF-7051800-I (5 bp del+) (Fig. 4a). The detachment assay also revealed that biofilms formed by the t9268 and t18946 isolates were susceptible to NMP, while the addition of proteinase K and DNase I had only minimal effects. This observation suggests that the biofilm mainly consists of an extracellular polysaccharide (EPS) and not PIA. The biofilm produced by t19031 *S. aureus* was equally dispersed by treatment with proteinase K, NMP, and DNase I (Fig. 4b).

### Cytotoxicity assay

**Fig. 5:**
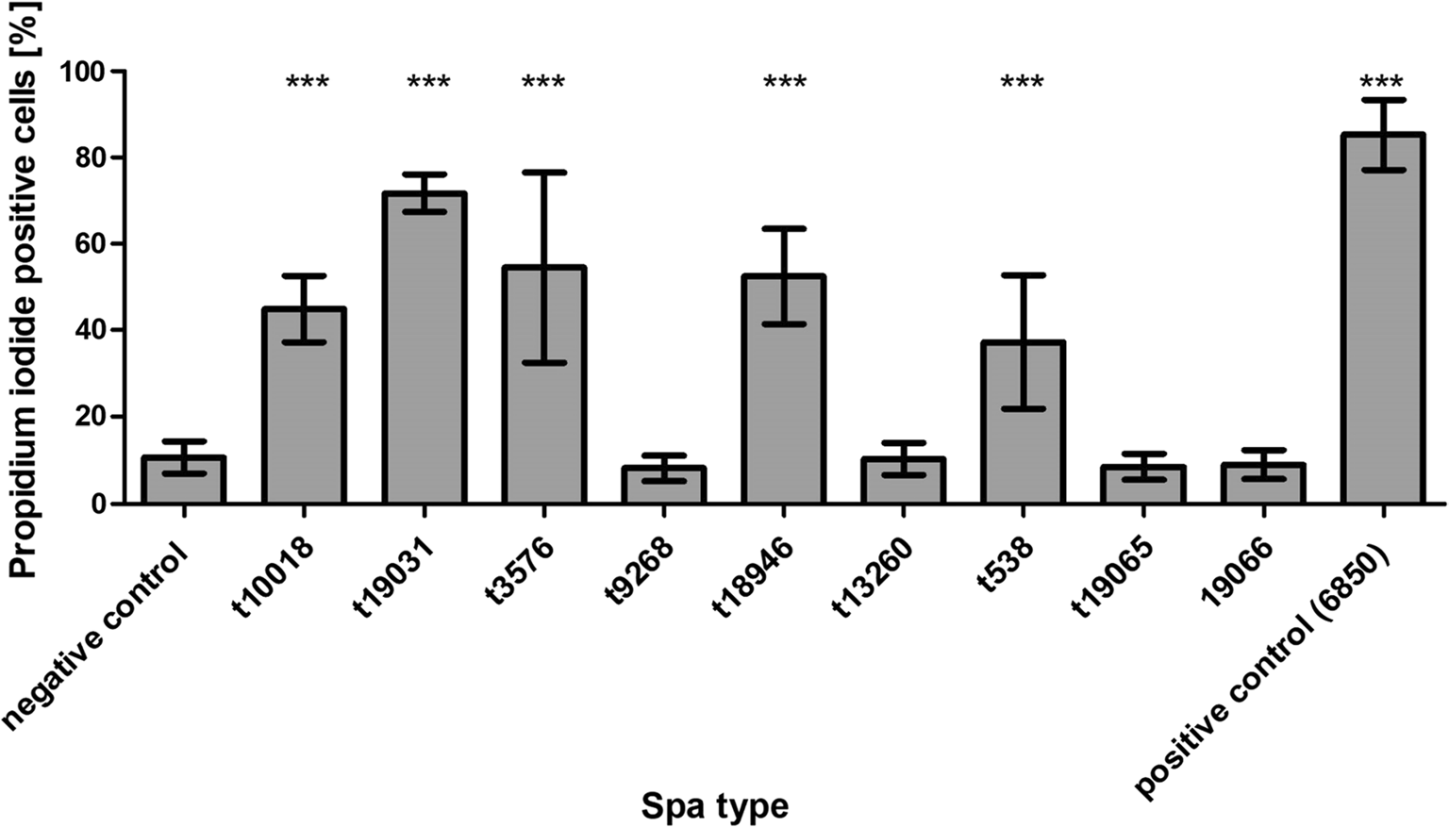
Extracellular cytotoxicity on A549 cells of *S. aureus* isolates representing *spa* types associated with CC522. Bars represent the mean and standard deviation of five independent experiments. Statistical significance: *p*-value < 0.0001 (***). Positive control: highly invasive and cytotoxic strain *S. aureus* 6850. Negative control: TSB broth

We observed that WGS detected less virulence (enterotoxins and toxic shock syndrome, leukocidins and hemolysins, exfoliative toxins) genes in CC522 *S. aureus* (median: 3; range 1–7) compared with CC133 (median: 10; range 9–11, Table 2). Thus, we explored the pathogenic potential of the CC522 lineage by investigating the cytotoxicity of secreted products of representative *S. aureus* on A549 cells. The assay showed that t538, t3576, t10018, t18946, t19031 isolates were strongly cytotoxic compared with the control (sterile TSB) (Fig. 5).

## Discussion

This study revealed a low prevalence of antibiotic-resistant *S. aureus* in the WAD goat in Nigeria. Only 4% (4/90) were identified as MRSA. Our observation is similar to previous goat studies in China [35], Iran [36], Nigeria [37], and Poland [38]. Penicillin and tetracycline are commonly used antibiotics in poultry and livestock farming in Nigeria [39, 40]. Our informal interaction with the goat sellers revealed that tetracycline and metronidazole are the common antibiotics administered to the ruminant animal. However, antibiotic use on the WAD goat is limited, which could be a plausible reason for the low percentage of antibiotic-resistant *S. aureus* observed in this study. Molecular typing and phylogenetic analysis also revealed that the *S. aureus* population of the WAD goat comprised primarily of CC133 and CC522 that are well adapted with ruminants. CC522 is one of the dominant *S. aureus* lineages among the goat population in China [35], Iran [36], Spain [41], and Tunisia [42]. It is also the main lineage in the anterior nares of healthy ewes in Tunisia [43]. The report of CC522 among the goat population in China and Iran suggests that this lineage may not be restricted to Africa and Europe, as postulated with sheep [44]. In this study, *S. aureus* assigned with t3576 and t10018 were the most common *spa* types and accounted for 64% (27/42) of the isolates in CC522. These genotypes have been identified from nasal samples of animals, including a farmworker (t10018) in Nigeria [37]. Besides, WGS showed that the t3576 and t10018 isolates were *tst*-positive (Table 2), which is similar to data from sheep [43] and dairy cows [45].

Although CC522 *S. aureus* was generally susceptible to all antibiotics and lacked many virulence genes based on WGS, the representative isolates demonstrated strong hemolytic activity and were confirmed as *hla* and *hlb*-positive. Moreover, t538, t3576, t10018, t18946, t19031 isolates showed strong cytotoxicity on A549 cells, unlike t9268, t13260, t19065, and t19066 *S. aureus* (Fig. 5). The reason for the variation is not clear. However, we postulate that strain specificity and level of expression of the hemolysins and/or other toxins [46] could be plausible reasons. Several methods have been employed for the detection and measurement of microbial biofilms [47]. In this study, the combination of touching single colonies with a sterile inoculating loop on CBA to assess the consistency and morphological characteristics on CRA was helpful in the presumptive identification of biofilm-producing *S. aureus*. We also demonstrated that CC522 *S. aureus* from the WAD goats could form biofilms. The semi-quantitative MTP and detachment assay provided evidence that the biofilm consists of an EPS and not PIA, as indicated by PCR and WGS. The nature of the EPS-associated biofilm is earmarked for further investigation. Overall, the ability of CC522 *S. aureus* to form biofilms, detection of *tst*, *hla*, *hlb*, and cytotoxic effect on A549 cells, regardless of their susceptibility to antibiotics, illustrate their pathogenic capability.

CC133 was the second most common group, which agrees with previous studies that it is frequently associated with ruminants [5, 44, 48–50]. There is evidence that CC133 could have evolved due to a human to ruminant host jump followed by adaptive genome diversification [51]. In this study, the *sec* and *tst* genes located on the *S. aureus* pathogenicity island *SaPIov1* were unique for the CC133 isolates, as previously noted [5, 50]. CC97, the third most common group, is a leading cause of bovine mastitis globally [52]. WGS showed that the isolates generally lacked the leukocidin and enterotoxin genes. However, PCR revealed that most of the CC97 isolates were *sak* + , suggesting a human host association.

The prevalence of human-associated lineages (CC1, CC5, CC8, CC15, CC30, CC45, CC121, and CC152) was lower (28%, 25/90) than animal-associated clones (68%, 65/90). *S. aureus* in CC1, CC30, CC121, and CC152 possessed the PVL genes, while the *lukDE* genes were identified in CC5, CC8, and CC15 (Table 2). PVL and LukDE are bi-component pore-forming leukocidins carried on the temperate phage ΦSa2 and *S. aureus* pathogenicity island vSaβ, respectively [53]. PVL-positive *S. aureus* is associated with subclinical mastitis in goats, while *lukDE* has a remarkable ability to target the lymphocytes of a broad host range [53]. Our observation of PVL-positive *S. aureus* from the WAD goat is similar to a study in China [35] and food-producing animals in Senegal [54]. PVL-positive *S. aureus* from colonized and clinical samples of humans has been widely reported in sub-Saharan Africa [55]. These findings indicate the possible impact of human-animal interaction on cross-species transmission.

MRSA has already been detected from the WAD goat in Nigeria [37, 56]. However, their molecular characteristics have not been well described. We identified MRSA with the following genotypes: t127-ST852-CC1-SCC*mec*VII, t4690-ST152-CC152-SCC*mec*Vc, and t8821-ST152-CC152-SCC*mec*Vc. ST852, a single locus variant (slv) of ST1, is the third most common MSSA clone associated with human infections in five major African towns [57]. It is also noteworthy that PVL-positive CC152-MRSA was recovered from the WAD goat. Although the CC152-MRSA lacked most of the enterotoxin and toxic shock genes, the isolates were positive for the hemolysins (*hla*, *hlb*, *hlgA*, *hlgB*) and epidermal differentiation factor B (*edinB*). WGS also revealed that the CC152 isolates (*scn*/*sak*-positive) possessed an intact *hlb* confirmed by PCR. This observation suggests new non-*hlb*-converting phages or alternative integration sites [29, 58]. The PVL-positive CC152 lineage is a successful MSSA clone among humans and animals in Africa [55, 59], including Nigeria [6, 12, 60]. The emergence of PVL-positive ST852/CC1 and ST152/CC152 MRSA indicates that it is important to understand the dynamics for introducing and acquiring the methicillin resistance (*mecA*) gene in these two successful African MSSA clones. Our work has limitations. First, we observed a poor agreement between WGS and conventional PCR in detecting some *S. aureus* antibiotic and virulence genes. Targets for WGS were considered as present if they were discovered in the genome with a range of ≥ 95% sequence identity and ≥ 99% query overlap to any of the sequences stored in the allele library [61]. Testing with appropriate and less restrictive threshold settings might provide increased concordance with PCR. Second, cultural and religious barriers could not allow us to investigate nasal and hand *S. aureus* carriage of close human contacts with the ruminant animal to address transmission and possible spread of antibiotic resistance genes. Social engagement and awareness on “One Health” with the evaluation of environmental samples are earmarked in subsequent investigations.

## Conclusion

Our study provides the first detailed analysis of the population structure and genomic content of *S. aureus* from the WAD goat in Nigeria. The *S. aureus* clonal population of the WAD goat is diverse, including new *spa* types and STs. This observation indicates limited existing data and the need for more surveillance studies on animal *S. aureus*. We present evidence that the *S. aureus* clonal population of the WAD goat consists of both ruminant-associated lineages, and human-associated clones. We also highlight the pathogenic potential of the antibiotic-susceptible and *tst*-positive CC522 *S. aureus*.

## Supplementary Information

**Additional file 1:Table S1.** Phenotypic and molecular characteristics of *Staphylococcus aureus* isolates from nasal samples of the WAD goat in Nigeria.

**Additional file 2:Table S2.** Percentage and level of agreement between antibiotic susceptibility testing (AST) and WGS with representative *S. aureus* isolates (n = 37) from the WAD goat in Nigeria..

**Additional file 3:Figure S3**. Distribution of spa-CC of *S. aureus* isolates from the WAD goat in Nigeria. Legend: Multilocus sequence typing (MLST) of the isolates was determined from WGS assembled files processed through the *S. aureus* (cg) MLST scheme. The sequence types (STs) were subsequently related to clonal complexes (CCs) using the eBURST algorithm. *new *spa* types.

**Additional file 4:Table S4.** Percentage and level of agreement between PCR and WGS in the detection of PVL and IEC genes from representative *S. aureus* isolates (n = 37) from the WAD goat in Nigeria.

**Additional file 5:Figure S5.** PCR detection of *hla* and *hlb* in *S. aureus* isolates representing various CCs. Legend: Gene and gene product: *hla*: 201bp; *hlb* 1+2: 534bp; *hlb* 2+3: 900bp; *hlb* 3+4: 140bp.

**Additional file 6:Table S6.** Characterization and detection of selected antibiotic resistance and virulence genes (WGS) in *S. aureus* isolates from the WAD goat in Nigeria.

## Data Availability

The data from the study is provided in Additional files.

## Abbreviations

ANOVA: Analysis of variance
AST: Antibiotic susceptibility testing
CBA: Columbia blood agar
CC: Clonal complex
cgMLST: Core genome multilocus sequence typing
chp: Chemotaxis-inhibiting protein
CRA: Congo red agar
DNase: Deoxyribonuclease
EPS: Extracellular polysaccharide
EUCAST: European committee on antimicrobial susceptibility testing
IEC: Immune evasion cluster
*lukF-PV*: Panton-valentine leukocidin subunit F
*lukS-PV*: Panton-valentine leukocidin subunit S
MALDI-TOF: Matrix assisted laser desorption ionization-time of flight
*mecA*: Methicillin resistance gene
MGEs: Mobile genetic elements
MRSA: Methicillin-resistant *Staphylococcus aureus*
MSCRAMM: Microbial surface components recognizing adhesive matrix molecules
MTP: Microtiter plate
NJ: Neighbor-joining
NMP: Sodium metaperiodate
NRPS: Nonribosomal peptide synthetase
PCR: Polymerase chain reaction
PIA: Polysaccharide intercellular adhesin
PI: Propidium iodide
PVL: Panton-Valentine leukocidin
sak: Staphylokinase
*S. aureus*: *Staphylococcus aureus*
SCC*mec*: Staphylococcal cassette chromosome *mec*
scn: Staphylococcal complement inhibitor
*Spa*: Staphylococcal protein A
ST: Sequence type
TSB: Tryptone soy broth
WAD: West African Dwarf
WGS: Whole-genome sequencing
