# Supplementary material for: Genomic analysis of Staphylococcus aureus from the West African Dwarf (WAD) goat in Nigeria
Source: Antimicrob Resist Infect Control. 2021 Aug 19;10:122. doi: 10.1186/s13756-021-00987-8 (PMC8375196; doi:10.1186/s13756-021-00987-8)
Supplement: Supplementary file 2 — Additional file 2:Table S2. Percentage and level of agreement between antibiotic susceptibility testing (AST) and WGS with representative S. aureus isolates (n = 37) from the WAD goat in Nigeria. [file 13756_2021_987_MOESM2_ESM.doc]

**Table S2** Percentage and level of agreement between antibiotic susceptibility testing (AST) and WGS with representative *S. aureus* isolates (n=37) from the WAD goat in Nigeria

| Erythromycin | | | |
| --- | --- | --- | --- |
| Antibiotic susceptibility testing | WGS (*ermA*) | | Total |
|  | Negative | Positive |  |
| Susceptible | 36 | 1 | 37 |
| Resistant | 0 | 0 | 0 |
| Total | 36 | 1 | 37 |
| Agreement (%) | 97% |  |  |
| Kappa coefficient | 0.00 | 95% CI: -0.00 to 0.00 |  |
| Fosfomycin | | | |
| Antibiotic susceptibility testing | WGS (*fosB*) | | Total |
|  | Negative | Positive |  |
| Susceptible | 24 | 13 | 37 |
| Resistant | 0 | 0 | 0 |
| Total | 24 | 13 | 37 |
| Agreement (%) | 65% |  |  |
| Kappa coefficient | 0.00 | 95% CI: -0.00 to 0.00 |  |
| Gentamicin | | | |
| Antibiotic susceptibility testing | WGS (*aacA-aphD, aadD, aphA3*) | | Total |
|  | Negative | Positive |  |
| Susceptible | 35 | 1 | 36 |
| Resistant | 0 | 1 | 1 |
| Total | 35 | 2 | 37 |
| Agreement (%) | 97% |  |  |
| Kappa coefficient | 0.65 | 95% CI: -0.03 to 1.00 |  |
| Oxacillin | | | |
| Antibiotic susceptibility testing | WGS (*mecA*) | | Total |
|  | Negative | Positive |  |
| Susceptible | 34 | 0 | 34 |
| Resistant | 0 | 3 | 3 |
| Total | 34 | 3 | 37 |
| Agreement (%) | 100% |  |  |
| Kappa coefficient | 1.00 | 95% CI: 1.00 to 1.00 |  |
| Penicillin | | | |
| Antibiotic susceptibility testing | WGS (*blaZ*) | |  |
|  | Negative | Positive | Total |
| Susceptible | 18 | 1 | 19 |
| Resistant | 4 | 14 | 18 |
| Total | 22 | 15 | 37 |
| Agreement (%) | 87% |  |  |
| Kappa coefficient | 0.73 | 95% CI: 0.51 to 0.95 |  |
| Tetracycline |  |  |  |
| Antibiotic susceptibility testing | WGS (*tetK*) | |  |
|  | Negative | Positive |  |
| Susceptible | 29 | 0 | 29 |
| Resistant | 3 | 5 | 8 |
| Total | 32 | 5 | 37 |
| Agreement (%) | 92% |  |  |
| Kappa coefficient | 0.72 | 95% CI: 0.44 to 1.00 |  |
