# Supplementary material for: Genomic analysis of Staphylococcus aureus from the West African Dwarf (WAD) goat in Nigeria
Source: Antimicrob Resist Infect Control. 2021 Aug 19;10:122. doi: 10.1186/s13756-021-00987-8 (PMC8375196; doi:10.1186/s13756-021-00987-8)
Supplement: Supplementary file 3 — Additional file 3:Figure S3. Distribution of spa-CC of S. aureus isolates from the WAD goat in Nigeria. Legend: Multilocus sequence typing (MLST) of the isolates was determined from WGS assembled files processed through the S. aureus (cg) MLST scheme. The sequence types (STs) were subsequently related to clonal complexes (CCs) using the eBURST algorithm. *new spa types. [file 13756_2021_987_MOESM3_ESM.pptx]

## Slide 1
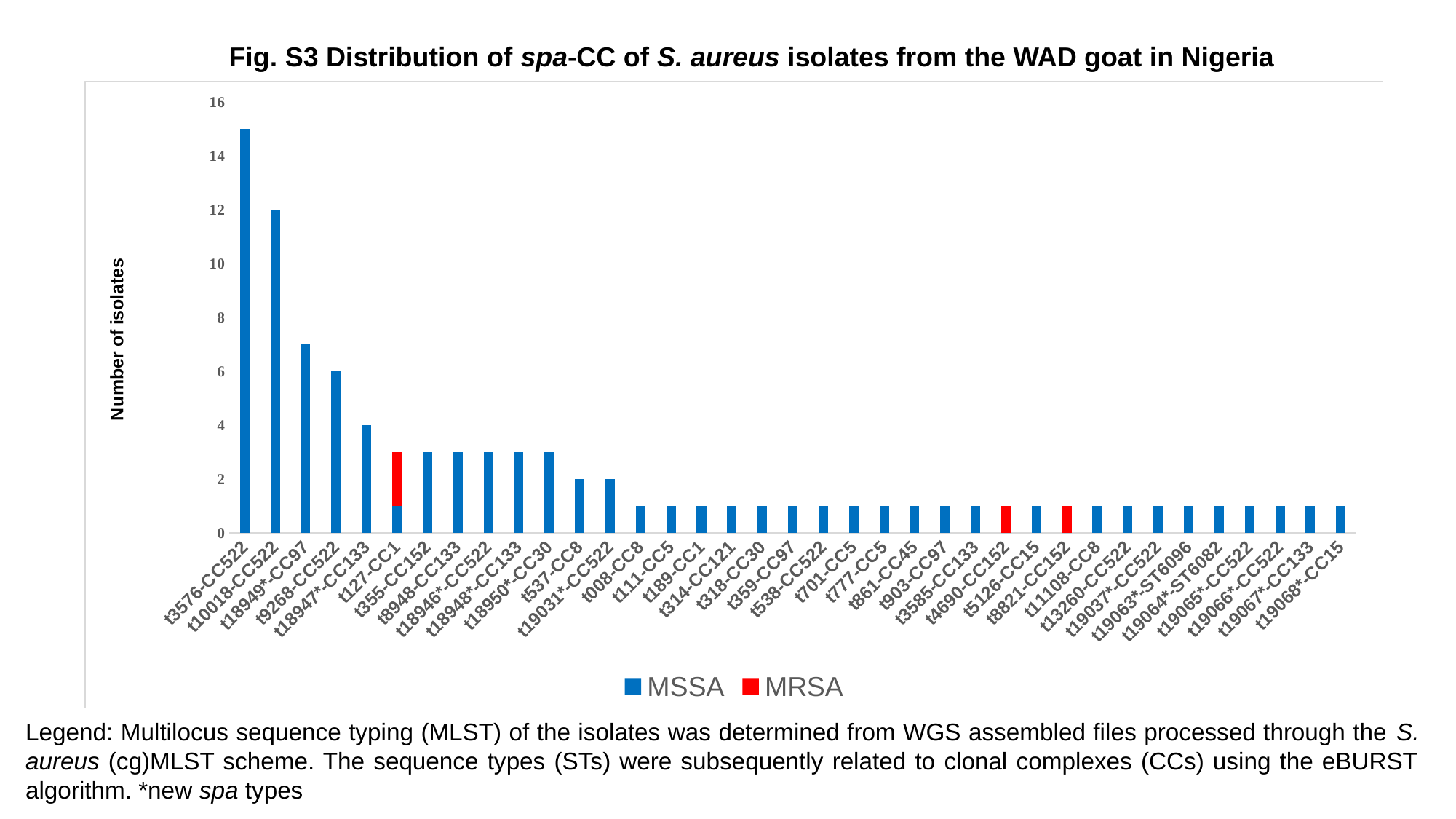

Fig. S3 Distribution of spa-CC of S. aureus isolates from the WAD goat in Nigeria
### Chart
| Category | | MSSA | MRSA |
|---|---|---|---|
| t3576-CC522 | None | 15.0 | 0.0 |
| t10018-CC522 | None | 12.0 | 0.0 |
| t18949*-CC97 | None | 7.0 | 0.0 |
| t9268-CC522 | None | 6.0 | 0.0 |
| t18947*-CC133 | None | 4.0 | 0.0 |
| t127-CC1 | None | 1.0 | 2.0 |
| t355-CC152 | None | 3.0 | 0.0 |
| t8948-CC133 | None | 3.0 | 0.0 |
| t18946*-CC522 | None | 3.0 | 0.0 |
| t18948*-CC133 | None | 3.0 | 0.0 |
| t18950*-CC30 | None | 3.0 | 0.0 |
| t537-CC8 | None | 2.0 | 0.0 |
| t19031*-CC522 | None | 2.0 | 0.0 |
| t008-CC8 | None | 1.0 | 0.0 |
| t111-CC5 | None | 1.0 | 0.0 |
| t189-CC1 | None | 1.0 | 0.0 |
| t314-CC121 | None | 1.0 | 0.0 |
| t318-CC30 | None | 1.0 | 0.0 |
| t359-CC97 | None | 1.0 | 0.0 |
| t538-CC522 | None | 1.0 | 0.0 |
| t701-CC5 | None | 1.0 | 0.0 |
| t777-CC5 | None | 1.0 | 0.0 |
| t861-CC45 | None | 1.0 | 0.0 |
| t903-CC97 | None | 1.0 | 0.0 |
| t3585-CC133 | None | 1.0 | 0.0 |
| t4690-CC152 | None | 0.0 | 1.0 |
| t5126-CC15 | None | 1.0 | 0.0 |
| t8821-CC152 | None | 0.0 | 1.0 |
| t11108-CC8 | None | 1.0 | 0.0 |
| t13260-CC522 | None | 1.0 | 0.0 |
| t19037*-CC522 | None | 1.0 | 0.0 |
| t19063*-ST6096 | None | 1.0 | 0.0 |
| t19064*-ST6082 | None | 1.0 | 0.0 |
| t19065*-CC522 | None | 1.0 | 0.0 |
| t19066*-CC522 | None | 1.0 | 0.0 |
| t19067*-CC133 | None | 1.0 | 0.0 |
| t19068*-CC15 | None | 1.0 | 0.0 |
Legend: Multilocus sequence typing (MLST) of the isolates was determined from WGS assembled files processed through the S. aureus (cg)MLST scheme. The sequence types (STs) were subsequently related to clonal complexes (CCs) using the eBURST algorithm. *new spa types
