# Supplementary material for: Genomic analysis of Staphylococcus aureus from the West African Dwarf (WAD) goat in Nigeria
Source: Antimicrob Resist Infect Control. 2021 Aug 19;10:122. doi: 10.1186/s13756-021-00987-8 (PMC8375196; doi:10.1186/s13756-021-00987-8)
Supplement: Supplementary file 4 — Additional file 4:Table S4. Percentage and level of agreement between PCR and WGS in the detection of PVL and IEC genes from representative S. aureus isolates (n = 37) from the WAD goat in Nigeria. [file 13756_2021_987_MOESM4_ESM.doc]

**Table S4 Percentage and level of agreement between PCR and WGS in the detection of PVL and IEC genes from representative *S. aureus* isolates (n=37) from the WAD goat in Nigeria**

| Panton-Valentine Leukocidin (PVL) | | | | |
| --- | --- | --- | --- | --- |
|  | | WGS | | Total |
|  |  | Negative | Positive |  |
| PCR | Negative | 30 | 0 | 30 |
|  | Positive | 4 | 3 | 7 |
|  | Total | 34 | 3 | 37 |
|  | Agreement (%) | 89% |  |  |
|  | Kappa coefficient | 0.55 | 95% CI: 0.18 to 0.92 |  |
| Chemotaxis inhibitory protein (Chp) | | | | |
|  | | WGS | | Total |
|  |  | Negative | Positive |  |
| PCR | Negative | 29 | 0 | 29 |
|  | Positive | 0 | 8 | 8 |
|  | Total | 29 | 8 | 37 |
|  | Agreement (%) | 100% |  |  |
|  | Kappa coefficient | 1.00 | 95%CI: 1.00 to 1.00 |  |
| Staphylokinase (Sak) | | | | |
|  | | WGS | |  |
|  |  | Negative | Positive | Total |
| PCR | Negative | 22 | 0 | 22 |
|  | Positive | 2 | 13 | 15 |
|  | Total | 24 | 13 | 37 |
|  | Agreement (%) | 95% |  |  |
|  | Kappa coefficient | 0.89 | 95%CI: 0.73 to 1.00 |  |
| Staphylococcal complement inhibitor (Scn) | | | | |
|  | | WGS | |  |
|  | | Negative | Positive |  |
| PCR | Negative | 21 | 0 | 21 |
|  | Positive | 0 | 16 | 16 |
|  | Total | 21 | 16 | 37 |
|  | Agreement (%) | 100% |  |  |
|  | Kappa coefficient | 1.000 | 95% CI: 1.00 to 1.00 |  |
