# Supplementary material for: Genomic analysis of Staphylococcus aureus from the West African Dwarf (WAD) goat in Nigeria
Source: Antimicrob Resist Infect Control. 2021 Aug 19;10:122. doi: 10.1186/s13756-021-00987-8 (PMC8375196; doi:10.1186/s13756-021-00987-8)
Supplement: Supplementary file 5 — Additional file 5:Figure S5. PCR detection of hla and hlb in S. aureus isolates representing various CCs. Legend: Gene and gene product: hla: 201bp; hlb 1+2: 534bp; hlb 2+3: 900bp; hlb 3+4: 140bp. [file 13756_2021_987_MOESM5_ESM.pptx]

## Slide 1
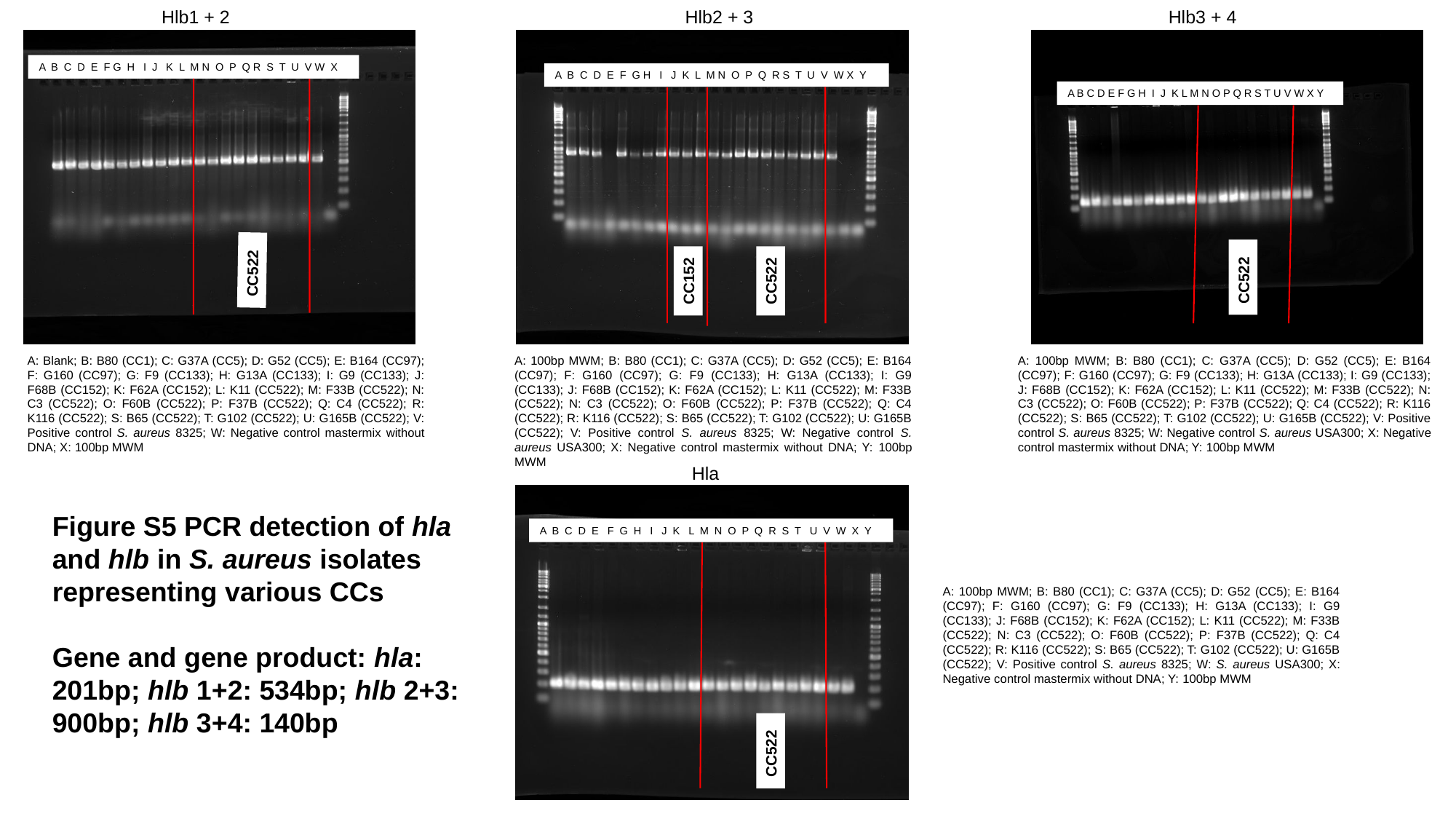

Hlb1 + 2
Hlb2 + 3
Hlb3 + 4
A B C D E F G H I J K L M N O P Q R S T U V W X
A B C D E F G H I J K L M N O P Q R S T U V W X Y
A B C D E F G H I J K L M N O P Q R S T U V W X Y
CC522
CC522
CC152
CC522
A: Blank; B: B80 (CC1); C: G37A (CC5); D: G52 (CC5); E: B164 (CC97); F: G160 (CC97); G: F9 (CC133); H: G13A (CC133); I: G9 (CC133); J: F68B (CC152); K: F62A (CC152); L: K11 (CC522); M: F33B (CC522); N: C3 (CC522); O: F60B (CC522); P: F37B (CC522); Q: C4 (CC522); R: K116 (CC522); S: B65 (CC522); T: G102 (CC522); U: G165B (CC522); V: Positive control S. aureus 8325; W: Negative control mastermix without DNA; X: 100bp MWM
A: 100bp MWM; B: B80 (CC1); C: G37A (CC5); D: G52 (CC5); E: B164 (CC97); F: G160 (CC97); G: F9 (CC133); H: G13A (CC133); I: G9 (CC133); J: F68B (CC152); K: F62A (CC152); L: K11 (CC522); M: F33B (CC522); N: C3 (CC522); O: F60B (CC522); P: F37B (CC522); Q: C4 (CC522); R: K116 (CC522); S: B65 (CC522); T: G102 (CC522); U: G165B (CC522); V: Positive control S. aureus 8325; W: Negative control S. aureus USA300; X: Negative control mastermix without DNA; Y: 100bp MWM
A: 100bp MWM; B: B80 (CC1); C: G37A (CC5); D: G52 (CC5); E: B164 (CC97); F: G160 (CC97); G: F9 (CC133); H: G13A (CC133); I: G9 (CC133); J: F68B (CC152); K: F62A (CC152); L: K11 (CC522); M: F33B (CC522); N: C3 (CC522); O: F60B (CC522); P: F37B (CC522); Q: C4 (CC522); R: K116 (CC522); S: B65 (CC522); T: G102 (CC522); U: G165B (CC522); V: Positive control S. aureus 8325; W: Negative control S. aureus USA300; X: Negative control mastermix without DNA; Y: 100bp MWM
Hla
Figure S5 PCR detection of hla and hlb in S. aureus isolates representing various CCs
Gene and gene product: hla: 201bp; hlb 1+2: 534bp; hlb 2+3: 900bp; hlb 3+4: 140bp
A B C D E F G H I J K L M N O P Q R S T U V W X Y
A: 100bp MWM; B: B80 (CC1); C: G37A (CC5); D: G52 (CC5); E: B164 (CC97); F: G160 (CC97); G: F9 (CC133); H: G13A (CC133); I: G9 (CC133); J: F68B (CC152); K: F62A (CC152); L: K11 (CC522); M: F33B (CC522); N: C3 (CC522); O: F60B (CC522); P: F37B (CC522); Q: C4 (CC522); R: K116 (CC522); S: B65 (CC522); T: G102 (CC522); U: G165B (CC522); V: Positive control S. aureus 8325; W: S. aureus USA300; X: Negative control mastermix without DNA; Y: 100bp MWM
CC522
