# Supplementary material for: Genomic analysis of Staphylococcus aureus from the West African Dwarf (WAD) goat in Nigeria
Source: Antimicrob Resist Infect Control. 2021 Aug 19;10:122. doi: 10.1186/s13756-021-00987-8 (PMC8375196; doi:10.1186/s13756-021-00987-8)
Supplement: Supplementary file 6 — Additional file 6:Table S6. Characterization and detection of selected antibiotic resistance and virulence genes (WGS) in S. aureus isolates from the WAD goat in Nigeria. [file 13756_2021_987_MOESM6_ESM.doc]

**Table S6** Characterization and detection of selected antibiotic resistance and virulence genes (WGS) in *S. aureus* isolates from the WAD goat in Nigeria

|  | | | | **Molecular typing** | | | | **Antibiotic resistance** | | | | | | | | | | | | **Enterotoxins & toxic shock syndrome toxin** | | | | | | | | | | | | | | | |
| --- | --- | --- | --- | --- | --- | --- | --- | --- | --- | --- | --- | --- | --- | --- | --- | --- | --- | --- | --- | --- | --- | --- | --- | --- | --- | --- | --- | --- | --- | --- | --- | --- | --- | --- | --- |
|  | Code  Name | Location | Antibiogram | *Spa* (number of isolates) | ST | CC | cap | *aac/aph* | *aadD* | *aphA3* | *blaZ* | *dfrA* | *ermA* | *fosB* | *lmrP* | *mecA* | *mprF* | *tetK* | *sea-sep* | | *sec* | *sed* | *seg* | *seh* | *sei* | *sej* | *sek* | *sel* | *sem* | *sen* | *seo* | *seq* | *ser* | *seu* | *tst* |
| 1 | K102 | Osogbo | Pen, Tet | t189 (1) | 188 | 1 | 8 |  |  |  |  |  |  |  |  |  |  |  |  | |  |  |  |  |  |  |  |  |  |  |  |  |  |  |  |
| 2 | G24 | Ile-Ife | Pen, Oxa, Tet | t127 (3) | 852 | 1 | 8 |  |  |  |  |  |  |  |  |  |  |  |  | |  |  |  |  |  |  |  |  |  |  |  |  |  |  |  |
| 3 | B80 | Ede | Pen | t19037 (1) | 2077 | 1 | 5 |  |  |  |  |  |  |  |  |  |  |  |  | |  |  |  |  |  |  |  |  |  |  |  |  |  |  |  |
| 4 | G37A | Ile-Ife | Susceptible to all antibiotics | t111 (1) | 6091 | 5 | 5 |  |  |  |  |  |  |  |  |  |  |  |  | |  |  |  |  |  |  |  |  |  |  |  |  |  |  |  |
| 5 | G52 | Ile-Ife | Susceptible to all antibiotics | t777 (1) | 6091 | 5 | 5 |  |  |  |  |  |  |  |  |  |  |  |  | |  |  |  |  |  |  |  |  |  |  |  |  |  |  |  |
| 6 | F45A | Modakeke | Pen | t701 (1) | 6093 | 5 | 8 |  |  |  |  |  |  |  |  |  |  |  |  | |  |  |  |  |  |  |  |  |  |  |  |  |  |  |  |
| 7 | B1 | Ede | Pen, Tet | t537 (2) | 72 | 8 | 5 |  |  |  |  |  |  |  |  |  |  |  |  | |  |  |  |  |  |  |  |  |  |  |  |  |  |  |  |
| 8 | C169 | Ejigbo | Pen | t11108 (1) | 72 | 8 | 5 |  |  |  |  |  |  |  |  |  |  |  |  | |  |  |  |  |  |  |  |  |  |  |  |  |  |  |  |
| 9 | B32 | Ede | Pen, Cot | t008 (1) | 6094 | 8 | 5 |  |  |  |  |  |  |  |  |  |  |  |  | |  |  |  |  |  |  |  |  |  |  |  |  |  |  |  |
| 10 | K107 | Osogbo | Pen, Cot | t5126 (1) | 15 | 15 | 8 |  |  |  |  |  |  |  |  |  |  |  |  | |  |  |  |  |  |  |  |  |  |  |  |  |  |  |  |
| 11 | C121 | Ejigbo | Pen, Tet, Cot | t19068 (1) | 15 | 15 | 8 |  |  |  |  |  |  |  |  |  |  |  |  | |  |  |  |  |  |  |  |  |  |  |  |  |  |  |  |
| 12 | K7B | Osogbo | Pen, Gen | t318 (1) | 30 | 30 | 8 |  |  |  |  |  |  |  |  |  |  |  |  | |  |  |  |  |  |  |  |  |  |  |  |  |  |  |  |
| 13 | G45 | Ile-Ife | Pen, Cot | t18950 (3) | 30 | 30 | 8 |  |  |  |  |  |  |  |  |  |  |  |  | |  |  |  |  |  |  |  |  |  |  |  |  |  |  |  |
| 14 | G157 | Ile-Ife | Pen | t861 (1) | 508 | 45 | 8 |  |  |  |  |  |  |  |  |  |  |  |  | |  |  |  |  |  |  |  |  |  |  |  |  |  |  |  |
| 15 | C8 | Ejigbo | Susceptible to all antibiotics | t18949 (7) | 97 | 97 | 5 |  |  |  |  |  |  |  |  |  |  |  |  | |  |  |  |  |  |  |  |  |  |  |  |  |  |  |  |
| 16 | B164 | Ede | Pen, Tet | t903 (1) | 6095 | 97 | 5 |  |  |  |  |  |  |  |  |  |  |  |  | |  |  |  |  |  |  |  |  |  |  |  |  |  |  |  |
| 17 | G160 | Ile-Ife | Susceptible to all antibiotics | t359 (1) | 6112 | 97 | 5 |  |  |  |  |  |  |  |  |  |  |  |  | |  |  |  |  |  |  |  |  |  |  |  |  |  |  |  |
| 18 | F12A | Modakeke | Pen, Cot | t314 (1) | 121 | 121 | 8 |  |  |  |  |  |  |  |  |  |  |  |  | |  |  |  |  |  |  |  |  |  |  |  |  |  |  |  |
| 19 | B110 | Ede | Susceptible to all antibiotics | t3585 (1) | 133 | 133 | 8 |  |  |  |  |  |  |  |  |  |  |  |  | |  |  |  |  |  |  |  |  |  |  |  |  |  |  |  |
| 20 | F9 | Modakeke | Susceptible to all antibiotics | t8948 (3) | 133 | 133 | 8 |  |  |  |  |  |  |  |  |  |  |  |  | |  |  |  |  |  |  |  |  |  |  |  |  |  |  |  |
| 21 | G13A | Ile-Ife | Susceptible to all antibiotics | t18948 (3) | 133 | 133 | 8 |  |  |  |  |  |  |  |  |  |  |  |  | |  |  |  |  |  |  |  |  |  |  |  |  |  |  |  |
| 22 | G169 | Ile-Ife | Pen, Cot | t19067 (1) | 133 | 133 | 8 |  |  |  |  |  |  |  |  |  |  |  |  | |  |  |  |  |  |  |  |  |  |  |  |  |  |  |  |
| 23 | G9 | Ile-Ife | Susceptible to all antibiotics | t18947 (4) | 133 | 133 | 8 |  |  |  |  |  |  |  |  |  |  |  |  | |  |  |  |  |  |  |  |  |  |  |  |  |  |  |  |
| 24 | F68B | Modakeke | Pen, Tet | t355 (3) | 152 | 152 | 5 |  |  |  |  |  |  |  |  |  |  |  |  | |  |  |  |  |  |  |  |  |  |  |  |  |  |  |  |
| 25 | F62A | Modakeke | Pen, Oxa, Tet, Cot | t4690 (1) | 152 | 152 | 5 |  |  |  |  |  |  |  |  |  |  |  |  | |  |  |  |  |  |  |  |  |  |  |  |  |  |  |  |
| 26 | K11 | Osogbo | Pen, Oxa, Tet, Cot | t8821 (1) | 152 | 152 | 5 |  |  |  |  |  |  |  |  |  |  |  |  | |  |  |  |  |  |  |  |  |  |  |  |  |  |  |  |
| 27 | F33B | Modakeke | Susceptible to all antibiotics | t9268 (6) | 522 | 522 | 5/8 |  |  |  |  |  |  |  |  |  |  |  |  | |  |  |  |  |  |  |  |  |  |  |  |  |  |  |  |
| 28 | C3 | Ejigbo | Susceptible to all antibiotics | t10018 (12) | 522 | 522 | 8 |  |  |  |  |  |  |  |  |  |  |  |  | |  |  |  |  |  |  |  |  |  |  |  |  |  |  |  |
| 29 | F60B | Modakeke | Susceptible to all antibiotics | t13260 (1) | 522 | 522 | 8 |  |  |  |  |  |  |  |  |  |  |  |  | |  |  |  |  |  |  |  |  |  |  |  |  |  |  |  |
| 30 | F37B | Modakeke | Susceptible to all antibiotics | t18946 (3) | 522 | 522 | 8 |  |  |  |  |  |  |  |  |  |  |  |  | |  |  |  |  |  |  |  |  |  |  |  |  |  |  |  |
| 31 | C4 | Ejigbo | Susceptible to all antibiotics | t19031 (2) | 522 | 522 | 8 |  |  |  |  |  |  |  |  |  |  |  |  | |  |  |  |  |  |  |  |  |  |  |  |  |  |  |  |
| 32 | K116 | Osogbo | Susceptible to all antibiotics | t19066 (1) | 522 | 522 | 8 |  |  |  |  |  |  |  |  |  |  |  |  | |  |  |  |  |  |  |  |  |  |  |  |  |  |  |  |
| 33 | B65 | Ede | Susceptible to all antibiotics | t3576 (15) | 2057 | 522 | 8 |  |  |  |  |  |  |  |  |  |  |  |  | |  |  |  |  |  |  |  |  |  |  |  |  |  |  |  |
| 34 | B161B | Ede | Susceptible to all antibiotics | t19064 (1) | 6082 | - | NT |  |  |  |  |  |  |  |  |  |  |  |  | |  |  |  |  |  |  |  |  |  |  |  |  |  |  |  |
| 35 | G102 | Ile-Ife | Susceptible to all antibiotics | t538 (1) | 6092 | 522 | 8 |  |  |  |  |  |  |  |  |  |  |  |  | |  |  |  |  |  |  |  |  |  |  |  |  |  |  |  |
| 36 | G165B | Ile-Ife | Susceptible to all antibiotics | t19065 (1) | 6092 | 522 | 8 |  |  |  |  |  |  |  |  |  |  |  |  | |  |  |  |  |  |  |  |  |  |  |  |  |  |  |  |
| 37 | B154 | Ede | Susceptible to all antibiotics | t19063 (1) | 6096 | - | NT |  |  |  |  |  |  |  |  |  |  |  |  | |  |  |  |  |  |  |  |  |  |  |  |  |  |  |  |

|  | | | | **Molecular typing** | | | | **Leukocidins & Haemolysins** | | | | | | | | | | | **Immune-evasion cluster** | | | **Exf. toxin & EDIN genes** | | | **Proteases** | | | | | | | **Intercell. adhesion genes** | | |
| --- | --- | --- | --- | --- | --- | --- | --- | --- | --- | --- | --- | --- | --- | --- | --- | --- | --- | --- | --- | --- | --- | --- | --- | --- | --- | --- | --- | --- | --- | --- | --- | --- | --- | --- |
|  | Code  Name | Location | Antibiogram | *Spa* (number of isolates) | ST | CC | cap | *lukD* | *lukE* | *lukF-PV* | *lukFP83* | *lukM* | *lukS-PV* | *hla* | *hlaB* | *hlgA* | *hlgB* | *hlgC* | *chp* | *sak* | *scn* | *etA* | *etD* | *edinB* | *aur* | *splA* | *splB* | *splE* | *sspA* | *sspB* | *sspP* | *icaA* | *icaC* | *icaD* |
| 1 | K102 | Osogbo | Pen, Tet | t189 (1) | 188 | 1 | 8 |  |  |  |  |  |  |  |  |  |  |  |  |  |  |  |  |  |  |  |  |  |  |  |  |  |  |  |
| 2 | G24 | Ile-Ife | Pen, Oxa, Te | t127 (3) | 852 | 1 | 8 |  |  |  |  |  |  |  |  |  |  |  |  |  |  |  |  |  |  |  |  |  |  |  |  |  |  |  |
| 3 | B80 | Ede | Pen | t19037 (1) | 2077 | 1 | 5 |  |  |  |  |  |  |  |  |  |  |  |  |  |  |  |  |  |  |  |  |  |  |  |  |  |  |  |
| 4 | G37A | Ile-Ife | Susceptible to all antibiotics | t111 (1) | 6091 | 5 | 5 |  |  |  |  |  |  |  |  |  |  |  |  |  |  |  |  |  |  |  |  |  |  |  |  |  |  |  |
| 5 | G52 | Ile-Ife | Susceptible to all antibiotics | t777 (1) | 6091 | 5 | 5 |  |  |  |  |  |  |  |  |  |  |  |  |  |  |  |  |  |  |  |  |  |  |  |  |  |  |  |
| 6 | F45A | Modakeke | Pen | t701 (1) | 6093 | 5 | 8 |  |  |  |  |  |  |  |  |  |  |  |  |  |  |  |  |  |  |  |  |  |  |  |  |  |  |  |
| 7 | B1 | Ede | Pen, Tet | t537 (2) | 72 | 8 | 5 |  |  |  |  |  |  |  |  |  |  |  |  |  |  |  |  |  |  |  |  |  |  |  |  |  |  |  |
| 8 | C169 | Ejigbo | Pen | t11108 (1) | 72 | 8 | 5 |  |  |  |  |  |  |  |  |  |  |  |  |  |  |  |  |  |  |  |  |  |  |  |  |  |  |  |
| 9 | B32 | Ede | Pen, Cot | t008 (1) | 6094 | 8 | 5 |  |  |  |  |  |  |  |  |  |  |  |  |  |  |  |  |  |  |  |  |  |  |  |  |  |  |  |
| 10 | K107 | Osogbo | Pen, Cot | t5126 (1) | 15 | 15 | 8 |  |  |  |  |  |  |  |  |  |  |  |  |  |  |  |  |  |  |  |  |  |  |  |  |  |  |  |
| 11 | C121 | Ejigbo | Pen, Tet, Cot | t19068 (1) | 15 | 15 | 8 |  |  |  |  |  |  |  |  |  |  |  |  |  |  |  |  |  |  |  |  |  |  |  |  |  |  |  |
| 12 | K7B | Osogbo | Pen, Gen | t318 (1) | 30 | 30 | 8 |  |  |  |  |  |  |  |  |  |  |  |  |  |  |  |  |  |  |  |  |  |  |  |  |  |  |  |
| 13 | G45 | Ile-Ife | Pen, Cot | t18950 (3) | 30 | 30 | 8 |  |  |  |  |  |  |  |  |  |  |  |  |  |  |  |  |  |  |  |  |  |  |  |  |  |  |  |
| 14 | G157 | Ile-Ife | Pen | t861 (1) | 508 | 45 | 8 |  |  |  |  |  |  |  |  |  |  |  |  |  |  |  |  |  |  |  |  |  |  |  |  |  |  |  |
| 15 | C8 | Ejigbo | Susceptible to all antibiotics | t18949 (7) | 97 | 97 | 5 |  |  |  |  |  |  |  |  |  |  |  |  |  |  |  |  |  |  |  |  |  |  |  |  |  |  |  |
| 16 | B164 | Ede | Pen, Tet | t903 (1) | 6095 | 97 | 5 |  |  |  |  |  |  |  |  |  |  |  |  |  |  |  |  |  |  |  |  |  |  |  |  |  |  |  |
| 17 | G160 | Ile-Ife | Susceptible to all antibiotics | t359 (1) | 6112 | 97 | 5 |  |  |  |  |  |  |  |  |  |  |  |  |  |  |  |  |  |  |  |  |  |  |  |  |  |  |  |
| 18 | F12A | Modakeke | Pen, Cot | t314 (1) | 121 | 121 | 8 |  |  |  |  |  |  |  |  |  |  |  |  |  |  |  |  |  |  |  |  |  |  |  |  |  |  |  |
| 19 | B110 | Ede | Susceptible to all antibiotics | t3585 (1) | 133 | 133 | 8 |  |  |  |  |  |  |  |  |  |  |  |  |  |  |  |  |  |  |  |  |  |  |  |  |  |  |  |
| 20 | F9 | Modakeke | Susceptible to all antibiotics | t8948 (3) | 133 | 133 | 8 |  |  |  |  |  |  |  |  |  |  |  |  |  |  |  |  |  |  |  |  |  |  |  |  |  |  |  |
| 21 | G13A | Ile-Ife | Susceptible to all antibiotics | t18948 (3) | 133 | 133 | 8 |  |  |  |  |  |  |  |  |  |  |  |  |  |  |  |  |  |  |  |  |  |  |  |  |  |  |  |
| 22 | G169 | Ile-Ife | Pen, Cot | t19067 (1) | 133 | 133 | 8 |  |  |  |  |  |  |  |  |  |  |  |  |  |  |  |  |  |  |  |  |  |  |  |  |  |  |  |
| 23 | G9 | Ile-Ife | Susceptible to all antibiotics | t18947 (4) | 133 | 133 | 8 |  |  |  |  |  |  |  |  |  |  |  |  |  |  |  |  |  |  |  |  |  |  |  |  |  |  |  |
| 24 | F68B | Modakeke | Pen, Tet | t355 (3) | 152 | 152 | 5 |  |  |  |  |  |  |  |  |  |  |  |  |  |  |  |  |  |  |  |  |  |  |  |  |  |  |  |
| 25 | F62A | Modakeke | Pen, Oxa, Tet, Cot | t4690 (1) | 152 | 152 | 5 |  |  |  |  |  |  |  |  |  |  |  |  |  |  |  |  |  |  |  |  |  |  |  |  |  |  |  |
| 26 | K11 | Osogbo | Pen, Oxa, Tet, Cot | t8821 (1) | 152 | 152 | 5 |  |  |  |  |  |  |  |  |  |  |  |  |  |  |  |  |  |  |  |  |  |  |  |  |  |  |  |
| 27 | F33B | Modakeke | Susceptible to all antibiotics | t9268 (6) | 522 | 522 | 5/8 |  |  |  |  |  |  |  |  |  |  |  |  |  |  |  |  |  |  |  |  |  |  |  |  |  |  |  |
| 28 | C3 | Ejigbo | Susceptible to all antibiotics | t10018 (12) | 522 | 522 | 8 |  |  |  |  |  |  |  |  |  |  |  |  |  |  |  |  |  |  |  |  |  |  |  |  |  |  |  |
| 29 | F60B | Modakeke | Susceptible to all antibiotics | t13260 (1) | 522 | 522 | 8 |  |  |  |  |  |  |  |  |  |  |  |  |  |  |  |  |  |  |  |  |  |  |  |  |  |  |  |
| 30 | F37B | Modakeke | Susceptible to all antibiotics | t18946 (3) | 522 | 522 | 8 |  |  |  |  |  |  |  |  |  |  |  |  |  |  |  |  |  |  |  |  |  |  |  |  |  |  |  |
| 31 | C4 | Ejigbo | Susceptible to all antibiotics | t19031 (2) | 522 | 522 | 8 |  |  |  |  |  |  |  |  |  |  |  |  |  |  |  |  |  |  |  |  |  |  |  |  |  |  |  |
| 32 | K116 | Osogbo | Susceptible to all antibiotics | t19066 (1) | 522 | 522 | 8 |  |  |  |  |  |  |  |  |  |  |  |  |  |  |  |  |  |  |  |  |  |  |  |  |  |  |  |
| 33 | B65 | Ede | Susceptible to all antibiotics | t3576 (15) | 2057 | 522 | 8 |  |  |  |  |  |  |  |  |  |  |  |  |  |  |  |  |  |  |  |  |  |  |  |  |  |  |  |
| 34 | B161B | Ede | Susceptible to all antibiotics | t19064 (1) | 6082 | - | NT |  |  |  |  |  |  |  |  |  |  |  |  |  |  |  |  |  |  |  |  |  |  |  |  |  |  |  |
| 35 | G102 | Ile-Ife | Susceptible to all antibiotics | t538 (1) | 6092 | 522 | 8 |  |  |  |  |  |  |  |  |  |  |  |  |  |  |  |  |  |  |  |  |  |  |  |  |  |  |  |
| 36 | G165B | Ile-Ife | Susceptible to all antibiotics | t19065 (1) | 6092 | 522 | 8 |  |  |  |  |  |  |  |  |  |  |  |  |  |  |  |  |  |  |  |  |  |  |  |  |  |  |  |
| 37 | B154 | Ede | Susceptible to all antibiotics | t19063 (1) | 6096 | - | NT |  |  |  |  |  |  |  |  |  |  |  |  |  |  |  |  |  |  |  |  |  |  |  |  |  |  |  |

|  | | | | **Molecular typing** | | | | **MSCRAMMs** | | | | | | | | | | | | | | | | | | |
| --- | --- | --- | --- | --- | --- | --- | --- | --- | --- | --- | --- | --- | --- | --- | --- | --- | --- | --- | --- | --- | --- | --- | --- | --- | --- | --- |
|  | Code  Name | Location | Antibiogram | *Spa* (number of isolates) | ST | CC | cap | *clfA* | *clfB* | *cna* | *ebh* | *ebpS* | *eno* | *fib* | *fnbA* | *fnbB* | *hysA* | *isaB* | *isdA* | *map* | *sasG* | *sdrC* | *sdrD* | *sdrE* | *sdrM* | *vwb* |
| 1 | K102 | Osogbo | Pen, Tet | t189 (1) | 188 | 1 | 8 |  |  |  |  |  |  |  |  |  |  |  |  |  |  |  |  |  |  |  |
| 2 | G24 | Ile-Ife | Pen, Oxa, Tet | t127 (3) | 852 | 1 | 8 |  |  |  |  |  |  |  |  |  |  |  |  |  |  |  |  |  |  |  |
| 3 | B80 | Ede | Pen | t19037 (1) | 2077 | 1 | 5 |  |  |  |  |  |  |  |  |  |  |  |  |  |  |  |  |  |  |  |
| 4 | G37A | Ile-Ife | Susceptible to all antibiotics | t111 (1) | 6091 | 5 | 5 |  |  |  |  |  |  |  |  |  |  |  |  |  |  |  |  |  |  |  |
| 5 | G52 | Ile-Ife | Susceptible to all antibiotics | t777 (1) | 6091 | 5 | 5 |  |  |  |  |  |  |  |  |  |  |  |  |  |  |  |  |  |  |  |
| 6 | F45A | Modakeke | Pen | t701 (1) | 6093 | 5 | 8 |  |  |  |  |  |  |  |  |  |  |  |  |  |  |  |  |  |  |  |
| 7 | B1 | Ede | Pen, Tet | t537 (2) | 72 | 8 | 5 |  |  |  |  |  |  |  |  |  |  |  |  |  |  |  |  |  |  |  |
| 8 | C169 | Ejigbo | Pen | t11108 (1) | 72 | 8 | 5 |  |  |  |  |  |  |  |  |  |  |  |  |  |  |  |  |  |  |  |
| 9 | B32 | Ede | Pen, Cot | t008 (1) | 6094 | 8 | 5 |  |  |  |  |  |  |  |  |  |  |  |  |  |  |  |  |  |  |  |
| 10 | K107 | Osogbo | Pen, Cot | t5126 (1) | 15 | 15 | 8 |  |  |  |  |  |  |  |  |  |  |  |  |  |  |  |  |  |  |  |
| 11 | C121 | Ejigbo | Pen, Tet, Cot | t19068 (1) | 15 | 15 | 8 |  |  |  |  |  |  |  |  |  |  |  |  |  |  |  |  |  |  |  |
| 12 | K7B | Osogbo | Pen, Gen | t318 (1) | 30 | 30 | 8 |  |  |  |  |  |  |  |  |  |  |  |  |  |  |  |  |  |  |  |
| 13 | G45 | Ile-Ife | Pen, Cot | t18950 (3) | 30 | 30 | 8 |  |  |  |  |  |  |  |  |  |  |  |  |  |  |  |  |  |  |  |
| 14 | G157 | Ile-Ife | Pen | t861 (1) | 508 | 45 | 8 |  |  |  |  |  |  |  |  |  |  |  |  |  |  |  |  |  |  |  |
| 15 | C8 | Ejigbo | Susceptible to all antibiotics | t18949 (7) | 97 | 97 | 5 |  |  |  |  |  |  |  |  |  |  |  |  |  |  |  |  |  |  |  |
| 16 | B164 | Ede | Pen, Tet | t903 (1) | 6095 | 97 | 5 |  |  |  |  |  |  |  |  |  |  |  |  |  |  |  |  |  |  |  |
| 17 | G160 | Ile-Ife | Susceptible to all antibiotics | t359 (1) | 6112 | 97 | 5 |  |  |  |  |  |  |  |  |  |  |  |  |  |  |  |  |  |  |  |
| 18 | F12A | Modakeke | Pen, Cot | t314 (1) | 121 | 121 | 8 |  |  |  |  |  |  |  |  |  |  |  |  |  |  |  |  |  |  |  |
| 19 | B110 | Ede | Susceptible to all antibiotics | t3585 (1) | 133 | 133 | 8 |  |  |  |  |  |  |  |  |  |  |  |  |  |  |  |  |  |  |  |
| 20 | F9 | Modakeke | Susceptible to all antibiotics | t8948 (3) | 133 | 133 | 8 |  |  |  |  |  |  |  |  |  |  |  |  |  |  |  |  |  |  |  |
| 21 | G13A | Ile-Ife | Susceptible to all antibiotics | t18948 (3) | 133 | 133 | 8 |  |  |  |  |  |  |  |  |  |  |  |  |  |  |  |  |  |  |  |
| 22 | G169 | Ile-Ife | Pen, Cot | t19067 (1) | 133 | 133 | 8 |  |  |  |  |  |  |  |  |  |  |  |  |  |  |  |  |  |  |  |
| 23 | G9 | Ile-Ife | Susceptible to all antibiotics | t18947 (4) | 133 | 133 | 8 |  |  |  |  |  |  |  |  |  |  |  |  |  |  |  |  |  |  |  |
| 24 | F68B | Modakeke | Pen, Tet | t355 (3) | 152 | 152 | 5 |  |  |  |  |  |  |  |  |  |  |  |  |  |  |  |  |  |  |  |
| 25 | F62A | Modakeke | Pen, Oxa, Tet, Cot | t4690 (1) | 152 | 152 | 5 |  |  |  |  |  |  |  |  |  |  |  |  |  |  |  |  |  |  |  |
| 26 | K11 | Osogbo | Pen, Oxa, Tet, Cot | t8821 (1) | 152 | 152 | 5 |  |  |  |  |  |  |  |  |  |  |  |  |  |  |  |  |  |  |  |
| 27 | F33B | Modakeke | Susceptible to all antibiotics | t9268 (6) | 522 | 522 | 5/8 |  |  |  |  |  |  |  |  |  |  |  |  |  |  |  |  |  |  |  |
| 28 | C3 | Ejigbo | Susceptible to all antibiotics | t10018 (12) | 522 | 522 | 8 |  |  |  |  |  |  |  |  |  |  |  |  |  |  |  |  |  |  |  |
| 29 | F60B | Modakeke | Susceptible to all antibiotics | t13260 (1) | 522 | 522 | 8 |  |  |  |  |  |  |  |  |  |  |  |  |  |  |  |  |  |  |  |
| 30 | F37B | Modakeke | Susceptible to all antibiotics | t18946 (3) | 522 | 522 | 8 |  |  |  |  |  |  |  |  |  |  |  |  |  |  |  |  |  |  |  |
| 31 | C4 | Ejigbo | Susceptible to all antibiotics | t19031 (2) | 522 | 522 | 8 |  |  |  |  |  |  |  |  |  |  |  |  |  |  |  |  |  |  |  |
| 32 | K116 | Osogbo | Susceptible to all antibiotics | t19066 (1) | 522 | 522 | 8 |  |  |  |  |  |  |  |  |  |  |  |  |  |  |  |  |  |  |  |
| 33 | B65 | Ede | Susceptible to all antibiotics | t3576 (15) | 2057 | 522 | 8 |  |  |  |  |  |  |  |  |  |  |  |  |  |  |  |  |  |  |  |
| 34 | B161B | Ede | Susceptible to all antibiotics | t19064 (1) | 6082 | - | NT |  |  |  |  |  |  |  |  |  |  |  |  |  |  |  |  |  |  |  |
| 35 | G102 | Ile-Ife | Susceptible to all antibiotics | t538 (1) | 6092 | 522 | 8 |  |  |  |  |  |  |  |  |  |  |  |  |  |  |  |  |  |  |  |
| 36 | G165B | Ile-Ife | Susceptible to all antibiotics | t19065 (1) | 6092 | 522 | 8 |  |  |  |  |  |  |  |  |  |  |  |  |  |  |  |  |  |  |  |
| 37 | B154 | Ede | Susceptible to all antibiotics | t19063 (1) | 6096 | - | NT |  |  |  |  |  |  |  |  |  |  |  |  |  |  |  |  |  |  |  |

KEY: Pen, Penicillin; Gen, Gentamicin; Oxa, Oxacillin; Tet, Tetracycline; Cot, Trimethoprim-sulphamethoxazole; *spa*, *Staphylococcus* protein A; ST, Sequence Type; CC, clonal complex; cap, capsule; Antibiotic resistance genes and product (*aacA-aphD*, bi-functional aminoglycoside phosphotransferase; *aadD*, aminoglycoside adenyltransferase; *aphA3*, aminoglycoside phosphotransferase; *blaZ*, β-lactamase; *dfrA*, dihydrofolate reductase; *ermA*, rRNA adenine N-6-methyl-transferase gene; *fosB*, fosfomycin resistance protein; *lmrP*, integral membrane transporter, group 1; *mecA*, alternate penicillin-binding 2a; *mprF*, lysylphosphotidylglycerol synthetase; *tetK*, tetracycline efflux protein variant K). Enterotoxins & toxic shock syndrome genes (*sea*-*sep*, enterotoxin A & P; *sec*, enterotoxin C; *sed*, enterotoxin D; *seg*, enterotoxin G; *seh*, enterotoxin H; *sei*, enterotoxin I; *sej*, enterotoxin J; *sek*, enterotoxin K; *sel*, enterotoxin L; *sem*, enterotoxin M; *sen*, enterotoxin N, *seo*, enterotoxin O; *seq*, enterotoxin Q; *ser*, enterotoxin R; *seu*, enterotoxin U; *tst*, toxic shock syndrome toxin). Leukocidin & haemolysin genes (*lukD*, leukocidin D; *lukE*, leukocidin E; *lukF-PV*, Panton-Valentine Leukocidin subunit F; *lukF-P83*, bovine Panton-Valentine Leukocidin subunit F; *lukM*, Leukocidin M, *lukS-PV*, Panton-Valentine Leukocidin subunit S; *hla*, haemolysin alpha; *hlaB*, haemolysin B; *hlgA*, haemolysin gamma component A; *hlgB*, haemolysin gamma component B; *hlgC*, haemolysin gamma component C). Immune evasion cluster genes (*chp*, chemotaxis-inhibiting protein; *sak*, staphylokinase; *scn*; staphylococcal complement inhibitor). Exfoliative toxin & epidermal cell differentiation genes (*etA*, exfoliative toxin A; *etD*, exfoliative toxin D, *edinB*, epidermal cell differentiation inhibitor B). Proteases (*aur*, aureolysin; *splA*, serine protease A; *splB*, serine protease B; *splE*, serine protease E; *sspA*, glutamyl endopeptidase; *sspB*, staphopain B; *sspP*, staphopain A). Biofilm associated genes (*icaA*, intercellular adhesion protein A; *icaC*, intercellular adhesion protein C; *icaD*, intercellular adhesion protein D). MSCRAMMs, microbial surface components recognizing adhesive matrix molecules (*clfA*, clumping factor A; *clfB*, clumping factor B; *cna*, collagen-binding adhesion; *ebh*, cell wall-associated fibronectin-binding protein; *ebpS*, cell surface elastin binding protein; *eno*, enolase; *fib*, fibrinogen binding protein; *fnbA*, fibronectin-binding protein A; *fnbB*, fibronectin-binding protein B; *hysA*, hyaluronate lyase; *isaB*, immunodominant antigen B; *map*, major histocompatibility complex class II analog protein; *sasG*, *Staphylococcus aureus* surface protein G; *sdrC*, serine aspartate repeat protein C; *sdrD*, serine aspartate repeat protein D; *sdrE*, serine aspartate repeat protein E; *sdrM*, multidrug efflux protein; *vwb*, “van Willebrand factor” binding protein) □ negative ■ positive.
